# Production of (2*S*)-sakuranetin from (2*S*)-naringenin in *Escherichia coli* by strengthening methylation process and cell resistance

**DOI:** 10.1016/j.synbio.2022.07.004

**Published:** 2022-08-03

**Authors:** Qiumeng Sun, Song Gao, Shiqin Yu, Pu Zheng, Jingwen Zhou

**Affiliations:** a Science Center for Future Foods, Jiangnan University, 1800 Lihu Road, Wuxi, Jiangsu, 214122, China; b Key Laboratory of Industrial Biotechnology, Ministry of Education and School of Biotechnology, Jiangnan University, 1800 Lihu Road, Wuxi, Jiangsu, 214122, China; c Engineering Research Center of Ministry of Education on Food Synthetic Biotechnology, Jiangnan University, 1800 Lihu Road, Wuxi, Jiangsu, 214122, China; d Jiangsu Province Engineering Research Center of Food Synthetic Biotechnology, Jiangnan University, Wuxi, 214122, China

**Keywords:** (2*S*)-Sakuranetin, Flavonoid 7-*O*-methyltransferases, Methylation, Cell tolerance, Metabolic engineering

## Abstract

(2*S*)-Sakuranetin is a 7-*O*-methylflavonoid that has anticancer, antiviral, and antimicrobial activities. Methylation process is involved in biosynthesizing (2*S*)-sakuranetin from (2*S*)-naringenin, in which *S*-adenosylmethionine (SAM) serves as the methyl donor. In this study, after methyl donor and substrate inhibition were identified as limiting factors for (2*S*)-sakuranetin biosynthesis, an efficient (2*S*)-sakuranetin-producing strain was constructed by enhancing methyl donor supply and cell tolerance to (2*S*)-naringenin. Firstly, PfOMT3 from *Perilla frutescens* was selected as the optimal flavonoid 7-O-methyltransferase (F7-OMT) for the conversion of (2*S*)-naringenin to (2*S*)-sakuranetin. Then, the methylation process was upregulated by regulating pyridoxal 5′-phosphate (PLP) content, key enzymes in methionine synthesis pathway, and the availability of ATP. Furthermore, genes that can enhance cell resistance to (2*S*)-naringenin were identified from molecular chaperones and sRNAs. Finally, by optimizing the fermentation process, 681.44 mg/L of (2*S*)-sakuranetin was obtained in 250-mL shake flasks. The titer of (2*S*)-sakuranetin reached 2642.38 mg/L in a 5-L bioreactor, which is the highest titer ever reported. This work demonstrates the importance of cofactor PLP in methylation process, and provides insights to biosynthesize other *O*-methylated flavonoids efficiently in *E*. *coli*.

## Graphical abstract

**Figure:**
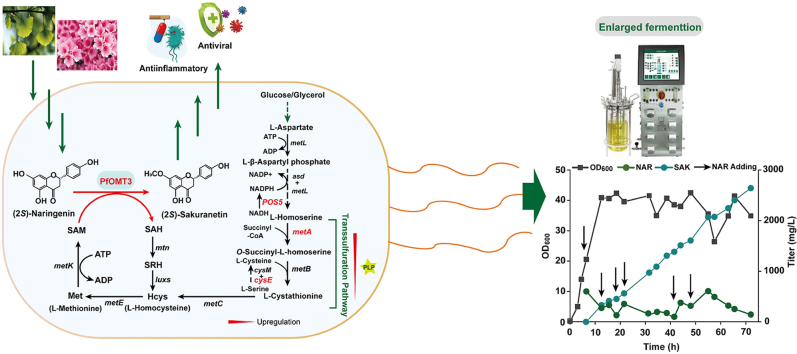

## 1 Introduction

(2*S*)-Sakuranetin (7-*O*-methylated (2*S*)-naringenin) is a 7-*O*-methylated flavonoid that is present in many plants, such as *Oryza sativa* [1], orange peel [2], and *Piper lanceifolium* Kunth [3]. It has been proved to have anti-inflammatory [4] (such as inhibiting 5ARII [5]), antimutation, anti-*Helicobacter pylori* [6], antidiabetic [7], antiviral (inhibiting viruses such as the influenza B/Lee/40 virus [8] and human rhinovirus 3 [9]), and anticonvulsant [10] properties. It can also exert protective effect on the brain, and can be used to treat Alzheimer's disease [11]. Considering the multiple biological effects of (2*S*)-sakuranetin, it has the potential to be used as nutraceutical or pharmaceutical agents.

(2*S*)-Sakuranetin is produced in plants in response to ambient pressures. For example, ultraviolet light or pest infection could activate 7-*O*-methyltransferase (7-OMT) in *Oryza sativa* to synthesize (2*S*)-sakuranetin [11]. Chemical synthesis and biosynthesis have been used for the synthesis of (2*S*)-sakuranetin. In a previous study, (2*S*)-sakuranetin was synthesized by chemically utilizing the regioselective deacetylation of naringenin triacetate [12]. However, this method is cumbersome and requires harsh reaction conditions. With the development of metabolic engineering and genetic engineering, (2*S*)-sakuranetin could be produced from (2*S*)-naringenin by flavonoid 7-*O*-methyltransferases (F7-OMTs) catalysis in *E*. *coli*. In a previous report, 40 mg/L of (2*S*)-sakuranetin was obtained by constructing a *de novo* biosynthesis pathway in *E. coli* [13]. 79 mg/L of (2*S*)-sakuranetin was produced in a 2.5-L bioreactor by using an *E. coli*–*E. coli* modular co-culture system [14].

F7-OMTs are *S*-adenosylmethionine (SAM)-dependent flavonoid methyltransferases that can transfer the methyl group from SAM to the 7-OH of flavonoids [6]. The limited supply of methyl donor in *E. coli* can restrict the accumulation of (2*S*)-sakuranetin. Previous studies focused on balancing the biosynthesis of (2*S*)-naringenin during the biosynthetic process of (2*S*)-sakuranetin, and there are no studies on strengthening the methylation of (2*S*)-naringenin to improve the production of (2*S*)-sakuranetin. In addition, flavonoids can interact with DNA, inhibit DNA gyrase [15] and damage the cytoplasmic membrane structure [16], which would interfere with the function of *E. coli*. Considering that microbes have developed certain mechanisms to adapt to environmental stress, various genetic strategies can be used to alleviate cell pressure, such as overexpression of molecular chaperones to refold the denatured proteins [17] and small regulatory RNAs (sRNAs) to alleviate stress [18,19]. Overexpression of molecular chaperones or sRNAs related to *E. coli* stress response might enhance cell tolerance to flavonoids so as to relieve substrate inhibition.

In this study, methylation process and substrate tolerance were enhanced to obtain (2*S*)-sakuranetin from (2*S*)-naringenin efficiently. Firstly, the optimal F-7OMT was selected. Then key enzymes (MetA, CysE) and cofactors (NADPH, ATP) essential for SAM biosynthesis were regulated to promote the methylation reaction. Moreover, the availability of an indispensable cofactor in the transsulfuration pathway, pyridoxal 5′-phosphate (PLP), was enhanced to increase the production of (2*S*)-sakuranetin. Additionally, sRNA RpoS was identified to be effective in enhancing cell tolerance to (2*S*)-naringenin. Finally, via the combinatorial expression of genes that were beneficial for (2*S*)-sakuranetin accumulation, and the optimization of fermentation process, 681.44 mg/L of (2*S*)-sakuranetin was obtained in shake-flask scale. The titer of (2*S*)-sakuranetin reached 2642.38 mg/L when scaled up in a 5-L bioreactor.

## 2 Materials and methods

### 2.1 Strains, plasmids, and genes

The strains used in this study are listed in Table 1. *E*. *coli* JM109 was used for plasmid propagation. *E*. *coli* BL21(DE3) was used for protein expression. The genes (*OsNOMT*, GenBank accession no. AB692949.1; *SaOMT2*, GenBank accession no. BAC70093; *PfOMT3*, GenBank accession no. MT909556) were synthesized by Sangon Biotech (Shanghai, China). The pET-28a (+), pACYCDuet-1 and pCDFDuet-1 expression vectors were obtained from Novagen (Darmstadt, Germany). High-fidelity Phusion DNA polymerase from Vazyme (Nanjing, China) was used for amplification. Seamless cloning kit purchased from Sangon Biotech (Shanghai) was used for plasmid assembly. The process for plasmids construction and primers are listed in the Supplementary File and Table S1, respectively.

**Table 1:** Strains used in the present study.

Strains	Description	Source
*E. coli* JM109	*recA1, endA1, gyrA96, thi-1, hsdR17* (*rk*^*-*^*mk*^*+*^)*, e14*^*-*^*(mcrA*^*-*^) *supE44, relA1, Δ* (*lac-proAB*)/F’[*traD36, proAB*^*+*^*, lacI^q^,* *lacZ*ΔM15]	Sangon Biotech (Shanghai)
*E. coli* BL21 (DE3)	F^-^*omp*T *hsd*S_B_ (rB^-^mB^-^) *gal dcmrne131*(DE3)	Sangon Biotech (Shanghai)
7-FOMT-1	*E. coli* BL21(DE3) carrying pET-28a (+)-*OsNOMT*	This study
7-FOMT-2	*E. coli* BL21(DE3) carrying pET-28a (+)-*SaOMT2*	This study
7-FOMT-3	*E. coli* BL21(DE3) carrying pET-28a (+)-*PfOMT3*	This study
NS01	*E. coli* BL21(DE3) carrying pCDFDuet-*metA-cysE*, pET-28a (+)-*PfOMT3*	This study
NS02	*E. coli* BL21(DE3) carrying pCDFDuet-*metA-cysE-metK*, pET-28a (+)-*PfOMT3*	This study
NS03	*E. coli* BL21(DE3) carrying pCDFDuet-*metA-cysE-ydaO*, pET-28a (+)-*PfOMT3*	This study
NS04	*E. coli* BL21(DE3) carrying pCDFDuet-*metA-cysE-ydaO-metK*, pET-28a (+)-*PfOMT3*	This study
NS07	*E. coli* BL21(DE3) carrying pACYCDuet-*SNZ3*, pET-28a (+)-*PfOMT3*	This study
NS08	*E. coli* BL21(DE3) carrying pACYCDuet-*RPS18B*, pET-28a (+)-*PfOMT3*	This study
NS09	*E. coli* BL21(DE3) carrying pACYCDuet-*RFC4*, pET-28a (+)-*PfOMT3*	This study
NS10	*E. coli* BL21(DE3) carrying pACYCDuet-*SNZ3-RPS18B*, pET-28a (+)-*PfOMT3*	This study
NS11	*E. coli BL21(DE3)* carrying pACYCDuet*-SNZ3-RPS18B-RFC4*, pET-28a (+)*-PfOMT3*	This study
NS14	*E. coli* BL21(DE3) carrying pET-28a (+)-*PfOMT3-POS5*	This study
NS15	*E. coli* BL21(DE3) carrying pET-28a (+)-*PfOMT3-POS5*, pCDFDuet-*metA-cysE-ydaO*	This study
NS16	*E. coli* BL21(DE3) carrying pET-28a (+)*-PfOMT3-POS5*, pACYCDuet*-SNZ3-RPS18B-RFC4*	This study
NS17	*E. coli* BL21(DE3) carrying pET-28a (+)*-PfOMT3*, pCDFDuet*-metA-cysE-ydaO*, pACYCDuet*-SNZ3*-*RPS18B-RFC4*	This study
NS18	*E. coli* BL21(DE3) carrying pET-28a (+)-*PfOMT3-POS5*, pCDFDuet-*metA-cysE-ydaO*, pACYCDuet-*SNZ3-RPS18B-RFC4*	This study
NS19	*E. coli* BL21(DE3) carrying pET-28a (+)-*PfOMT3-rpsL*	This study
NS20	*E. coli* BL21(DE3) carrying pET-28a (+)-*PfOMT3-rpsQ*	This study
NS21	*E. coli* BL21(DE3) carrying pET-28a (+)-*PfOMT3-rpsQ*^His31Pro^	This study
NS22	*E. coli* BL21(DE3) carrying pET-28a (+)-*PfOMT3-crp*	This study
NS23	*E. coli* BL21(DE3) carrying pET-28a (+)-*PfOMT3-rpoS*	This study
NS24	*E. coli* BL21(DE3) carrying pET-28a (+)-*PfOMT3-secB*	This study
NS25	*E. coli* BL21(DE3) carrying pET-28a (+)-*PfOMT3-nfuA*	This study
NS26	*E. coli* BL21(DE3) carrying pET-28a (+)-*PfOMT3-yajL*	This study
NS27	*E. coli* BL21(DE3) carrying pET-28a (+)-*PfOMT3-ycdy*	This study
NS28	*E. coli* BL21(DE3) carrying pET-28a (+)-*PfOMT3-proQ*	This study
NS29	*E. coli* BL21(DE3) carrying pET-28a (+)-*PfOMT3-nusB*	This study
NS30	*E. coli* BL21(DE3) carrying pET-28a (+)-*PfOMT3-acrR*	This study
NS31	*E. coli* BL21(DE3) carrying pET-28a (+)-*PfOMT3-asr*	This study
NS32	*E. coli* BL21(DE3) carrying pET-28a (+)-*PfOMT3-rpsQ*^His31Pro^*-rpoS*	This study
NS33	*E. coli* BL21(DE3) carrying pET-28a (+)-*PfOMT3-secB-rpoS*	This study
NS34	*E. coli* BL21(DE3) carrying pET-28a (+)-*PfOMT3-rpoS,* pCDFDuet-*metA-cysE-ydaO*, pACYCDuet-*SNZ3-RPS18B-RFC4*	This study
NS35	*E. coli* BL21(DE3) carrying pET-28a (+)-*PfOMT3-secB-rpoS,* pCDFDuet-*metA-cysE-ydaO,* pACYCDuet-*SNZ3-RPS18B-RFC4*	This study

### 2.2 Growth media and shake flask culture condition

The Luria–Bertani (LB) medium was used for culturing seed liquid. The Terrific Broth (TB) medium was used for (2*S*)-sakuranetin accumulation. Strains were cultured at 37 °C for 10 h in LB medium. Then a 2% (v/v) of LB strain culture was inoculated into the TB medium with shaking at 220 rpm. Isopropyl beta-D-thiogalactoside (IPTG) was added to the medium at a final concentration of 0.1 mM when OD_600_ reached 0.8–1, at which time the temperature was shifted from 37 °C to 25 °C. 350 mg/L of (2*S*)-naringenin was added after adding IPTG for 3 h and 13 h to a final concentration of 700 mg/L. The total fermentation time in shake-flask scale was 26 h.

### 2.3 Fermentation condition in bioreactor

Bioreactor fermentation was conducted in a 5-L glass bioreactor (T&J Bioengineering, Shanghai, China) containing 2.5 L of TB medium. The (2*S*)-naringenin was dissolved in methanol to a concentration of 50 g/L. The optimal strain was selected for scale-up culture in a 5-L bioreactor. Colonies from the culture plate were inoculated into LB liquid medium and incubated at 37 °C with shaking at 220 rpm for 10 h. Then, 4% (v/v) inoculum of seed culture was inoculated into a 5 L bioreactor containing 2.5 L of the TB medium. In the early stage, the fermentation temperature was kept at 37 °C and the rotating speed was 400 rpm. IPTG was added at a final concentration of 0.1 mM when the OD_600_ reached 6, at which time the temperature was shifted to 25 °C and the stirring speed was changed to the pattern of depending on dissolved oxygen content (40%). (2*S*)-Naringenin was fed in batches at 6.5 h, 12.5 h, 18.5 h, 21.5 h, 41.5 h, and 48 h to a final concentration of 4 g/L. 500 g/L of glycerol was fed at a speed of 8 mL/h after substrate was added, and 6 mM Mg^2+^ was added at 6.5 h. The pH was maintained at 7 ± 0.1 with 6 M NaOH during the fermentation.

### 2.4 Analytical methods

To quantitatively analyze the titer of (2*S*)-sakuranetin, a 500 μL fermentation sample was mixed with 500 μL methanol. Then, the supernatant was filtered using a 0.22-μm organic phase filter membrane after 12000g centrifugation for 5 min. The samples were detected by HPLC (Shimadzu Corporation, Japan) equipped with a Thermo Fisher C18 column (4.6 mm × 250 mm, 5 μm) at 290 nm. The mobile phase was as follows: A phase was water containing 0.1% TFA, and B phase was acetonitrile containing 0.1% TFA. The flow rate was 1 mL/min. The following procedure was used: 0–10 min, 10–60% B; 10–20 min, 40–80% B; 20–25 min, 80–10% B. The determination of the intracellular PLP level followed the procedure described by Cabo et al. [20].

### 2.5 Spot assays to assess *E. coli* substrate tolerance

*E. coli* only harboring *PfOMT3* and *E. coli* harboring cell tolerance genes were cultured at 37 °C in TB medium with shaking at 220 rpm. IPTG was added to the medium at a final concentration of 0.1 mM when OD_600_ reached 0.8. After 10 h post-induction at 25 °C, seeds were diluted by gradient (10^−2^, 10^−3^, 10^−4^, 10^−5^), and 2 μL of seeds dilution were spotted on solid LB plates containing (2*S*)-naringenin (200 mg/L, 300 mg/L or 400 mg/L). These LB plates were then incubated for 12 h at 37 °C.

## 3 Results

### 3.1 Effects of different sources of F7-OMTs on (2*S*)-sakuranetin production

Previous studies have confirmed that OsNOMT [21], SaOMT2 [22] and PfOMT3 [6] belong to the F7-OMTs family, and they have catalytic activity for converting (2*S*)-naringenin to (2*S*)-sakuranetin. In order to select the optimal F7-OMT, the production of (2*S*)-sakuranetin at different (2*S*)-naringenin concentrations with strains expressing F7-OMTs were compared. When 300 mg/L (2*S*)-naringenin was added after cells were induced for 10 h at 25 °C, 26.76 mg/L, 44.54 mg/L and 58.95 mg/L of (2*S*)-sakuranetin was achieved by strains harboring OsNOMT, SaOMT2 and PfOMT3, respectively (Fig. 2A), and PfOMT3 had better performance at other concentrations of (2*S*)-naringenin. Therefore, PfOMT3 was chosen for the following experiments. The strain expressing *PfOMT3* is referred as strain 7-FOMT-3. (2*S*)-Sakuranetin titer of strain 7-FOMT-3 showed a decreasing trend with the increase of (2*S*)-naringenin concentrations, and (2*S*)-sakuranetin titer decreased sharply when (2*S*)-naringenin concentration exceeded 250 mg/L (Fig. 2B). To remove the adverse impact brought by high concentration of the substrate, 250 mg/L of (2*S*)-naringenin was added in methylation enhancement experiments.

### 3.2 Regulation of methionine and ATP contents to improve (2*S*)-sakuranetin production

**Fig. 1:**
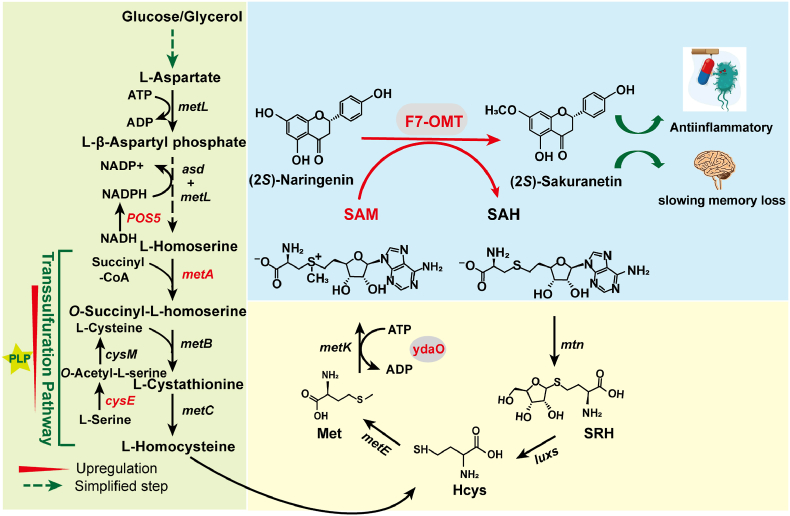
Biosynthesis pathway of (2*S*)-sakuranetin from (2*S*)-naringenin. The biosynthesis pathway of (2*S*)-sakuranetin from (2*S*)-naringenin involves a methylation reaction, where SAM (*S*-adenosylmethionine) is used as the methyl donor. SAM biosynthesis involves methionine metabolism and the regeneration of cofactors ATP, NADPH, and PLP. Met represents l-Methionine; Hcys represents l-Homocysteine; SRH represents *S*-Ribosyl-l-homocysteine; SAH reprsents *S*-Adenosyl-L-homocysteine.

Methyl donor SAM is involved in the conversion of (2*S*)-naringenin to (2*S*)-sakuranetin (Fig. 1). Exogenously supply of methionine can enter into cells and improve the intracellular SAM availability [23]. To test the importance of methyl donor in the biosynthesis of (2*S*)-sakuranetin, 250 mg/L (2*S*)-naringenin and different concentrations of methionine were added after cells were induced for 10 h at 25 °C (Fig. 3A). When 1 g/L methionine was added, 129.21 mg/L of (2*S*)-sakuranetin was obtained after reaction for 10 h, which was 36.17% higher than that of the strain 7-FOMT-3. In order to enhance methionine production of *E. coli*, *metA* (encoding homoserine succinyltransferase) and *cysE* (encoding l-serine *O*-acetyltransferase) were overexpressed (strain NS01). (2*S*)-Sakuranetin titer of strain NS01 reached 142.59 mg/L without the addition of methionine, which was 50.26% higher than the strain 7-FOMT-3. When *metK* (encoding SAM synthetase) was overexpressed in strain NS01, 135.26 mg/L of (2*S*)-sakuranetin was obtained, a slight decrease compared with the strain NS01.

**Fig. 2:**
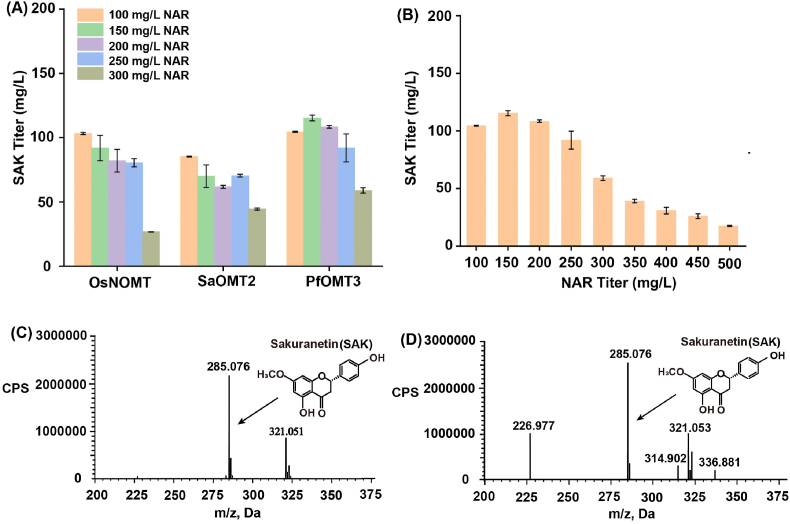
Effects of different sources of F7-OMTs on the titer of (2*S*)-sakuranetin. (A) The (2*S*)-sakuranetin production of F7-OMTs under different concentrations of (2*S*)-naringenin. (B) Summary of the production of (2*S*)-sakuranetin with strain 7-FOMT-3 at different (2*S*)-naringenin concentrations. (C) LC-MS analysis of the fermentation simple. (D) LC-MS analysis of the (2*S*)-sakuranetin standard simple. SAK represents (2*S*)-sakuranetin; NAR represents (2*S*)-naringenin.

**Fig. 3:**
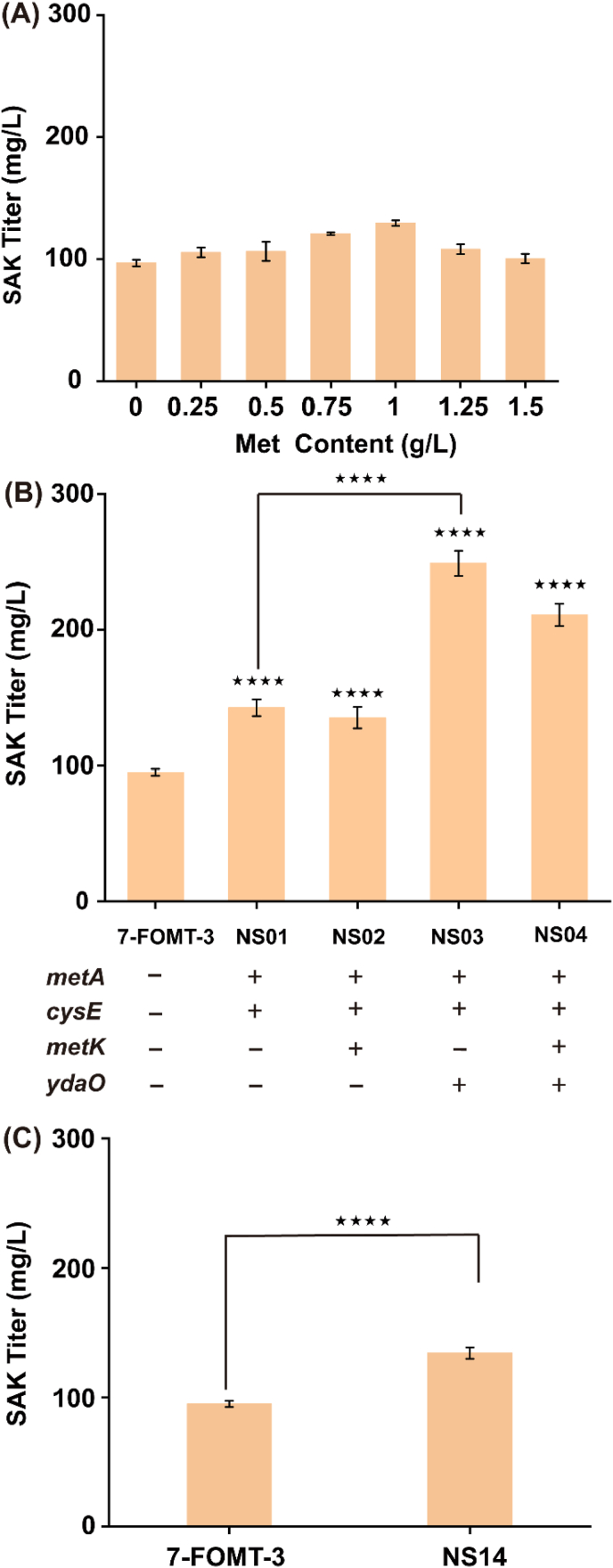
Effects of methionine, ATP and NADPH on (2*S*)-sakuranetin production. (A) (2*S*)-Sakuranetin titer at different methionine concentrations. (B) Effects of *metA*, *cysE*, *ydaO* and *metK* expression on (2*S*)-sakuranetin production. (C) Effect of *POS5* expression on (2*S*)-sakuranetin production. SAK represents (2*S*)-sakuranetin; NAR represents (2*S*)-naringenin; Met represents methionine. ****P < 0.0001.

ATP is consumed in the conversion of methionine to SAM, and is an important cofactor in methionine biosynthesis pathway. *ydaO*, which can dynamically regulate ATP accumulation in bacteria, was heterologously expressed in strain NS01 and NS02, resulting in strain NS03 and NS04, respectively; the (2*S*)-sakuranetin titer increased by 62.38% and 124.45%, respectively, compared with the strain 7-FOMT-3 (Fig. 3B). NADPH is another important cofactor in the methionine biosynthesis pathway. The NADH kinase-encoding gene *POS5* from *S. cerevisiae* was heterogeneously expressed in strain 7-FOMT-3 (resulting in strain NS14), improving the titer of (2*S*)-sakuranetin to 132.58 mg/L, which was 43.35% higher than that of the strain 7-FOMT-3 (Fig. 3C). These results suggested that the SAM pool is a limiting factor for the conversion of (2*S*)-naringenin to (2*S*)-sakuranetin. Regulating the levels of methionine and ATP are essential for (2*S*)-sakuranetin production.

### 3.3 Enhancement of PLP content to improve (2*S*)-sakuranetin production

**Fig. 4:**
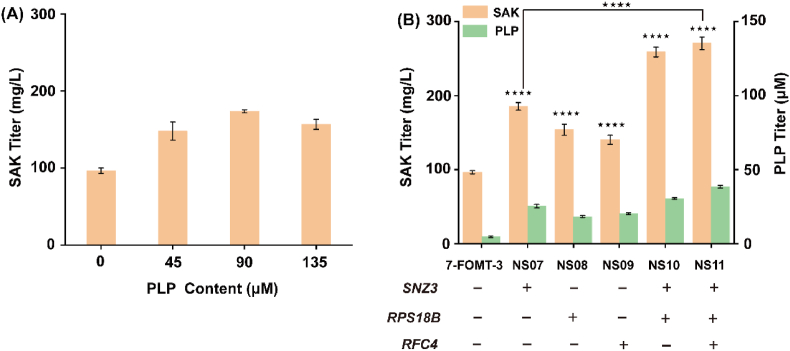
Effects of PLP on (2*S*)-sakuranetin production. (A) Effects of different concentrations of PLP on (2*S*)-sakuranetin accumulation. (B) Effects of *SNZ3*, *RFC4* and *RPS18B* expression on PLP and (2*S*)-sakuranetin titer. ****P < 0.0001. *SNZ3* encoding pyridoxal-5′-phosphate synthase; *RFC4* is a DNA binding protein; *RPS18B* is the component of the ribosomal subunit, which is homologous to *E*. *coli* ribosomal protein S13.

PLP is known to be a cofactor in the transsulfuration pathway [24]. To verify whether an increased PLP level enhances (2*S*)-sakuranetin production, 250 mg/L (2*S*)-naringenin and different concentrations of PLP were added after cells were induced for 10 h at 25 °C. The result showed that 173.61 mg/L (2*S*)-sakuranetin was obtained when 90 μM PLP was added, which increased by 79.85% compared with the group without PLP (Fig. 4A). In order to enhance the supply of PLP in *E. coli*, *SNZ3*, which encodes PLP synthase in *S. cerevisiae*, was expressed in strain 7-FOMT-3; (2*S*)-sakuranetin titer reached 185.35 mg/L without the addition of PLP, which increased by 92.39% compared with strain 7-FOMT-3. In addition, *RFC4* and *RPS18B* from *S. cerevisiae* [25] are also involved in PLP production. The (2*S*)-sakuranetin titer of strain NS06 (expressing *RFC4* in strain 7-FOMT-3) and NS07 (expressing *RPS18B* in strain 7-FOMT-3) increased by 59.59% and 45.69%, respectively, compared with the strain 7-FOMT-3. Then *SNZ3*, *RFC4* and *RPS18B* were co-expressed in strain 7-FOMT-3, resulting in strain NS11; the titer of (2*S*)-sakuranetin reached 260.45 mg/L, which was 170.34% higher than that of the strain 7-FOMT-3 (Fig. 4B), and the intracellular PLP level increased by 4.85-fold compared with strain 7-FOMT-3. Therefore, an increased PLP content is beneficial for (2*S*)-sakuranetin production.

### 3.4 Effects of combinatorial expression of methylation-enhancing genes on (2*S*)-sakuranetin production

**Fig. 5:**
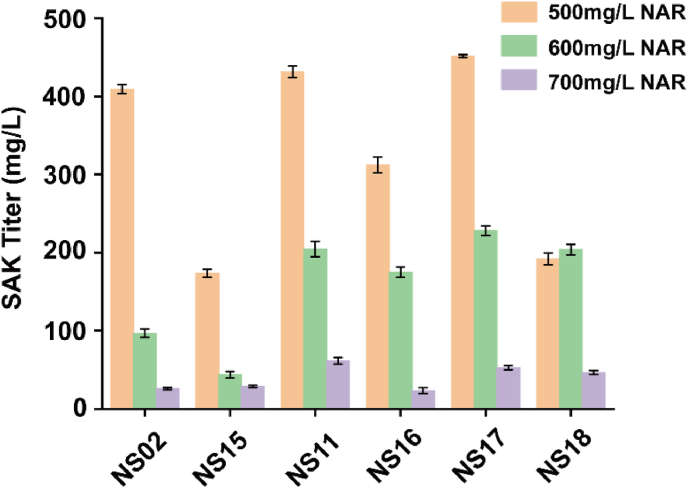
Selecting the optimal genes combination for enhancing methylation process. Effect of combinatorial expression of genes that can upregulate methylation reaction on (2*S*)-sakuranetin production. The (2*S*)-naringenin was added in batches.

Genes that can upregulate methylation reaction were expressed by combination. To avoid the negative effect of high concentration of (2*S*)-naringenin and obtain a higher titer of (2S)-sakuranetin, (2*S*)-naringenin was added in batches (final concentration was 500 mg/L, 600 mg/L, and 700 mg/L, respectively), in which the half quantity of (2*S*)-naringenin was added after adding IPTG for 1 h, and another 50% of (2*S*)-naringenin was added after 10 h. The result showed that strain NS17 was the best to accumulate (2*S*)-sakuranetin, and 451.47 mg/L of (2*S*)-sakuranetin was obtained when added 500 mg/L (2S)-naringenin (Fig. 5). When *POS5* was expressed with *PfOMT3* (strain NS14), this could promote the production of (2*S*)-sakuranetin, but (2*S*)-sakuranetin titer decreased when *POS5* was co-expressed with genes that enhanced the methylation reaction. Strain NS18 (expressing *POS5* in NS17) accumulated only 191.56 mg/L of (2*S*)-sakuranetin when added 500 mg/L (2*S*)-naringenin, resulting in a 57.57% decrease compared with the strain NS17. Based on the (2*S*)-sakuranetin titer, genes combination in strain NS17 was the best for strengthening the methylation process.

### 3.5 Enhancement of strain substrate tolerance to improve (2*S*)-sakuranetin production

**Fig. 6:**
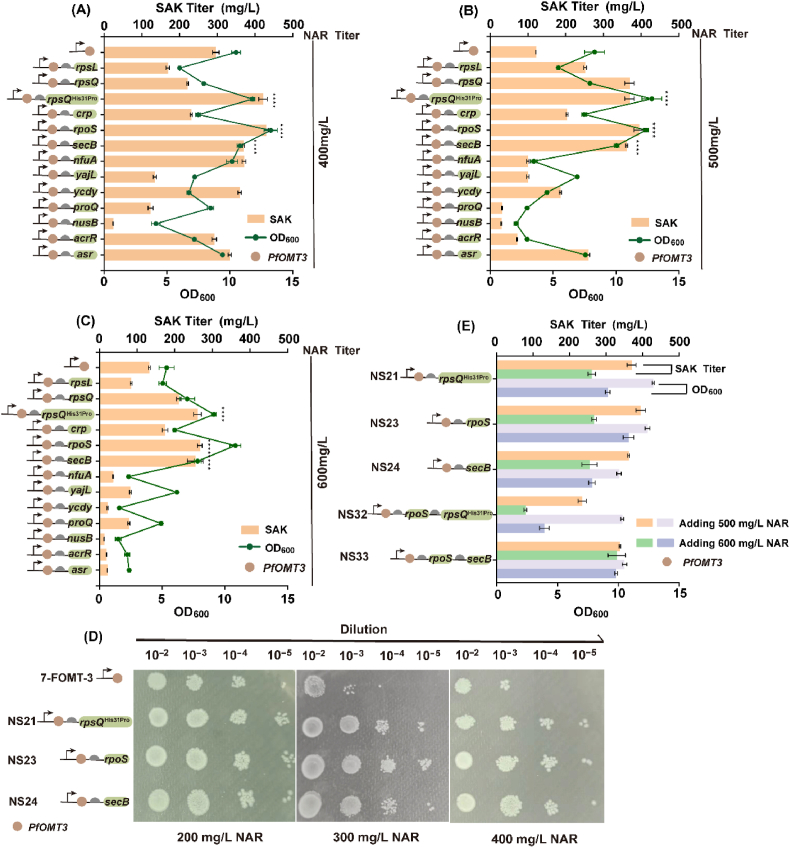
Improving substrate tolerance of strain to improve (2*S*)-sakuranetin production. (A) Effects of overexpressing stress resistance genes on (2*S*)-sakuranetin production and cell growth when 400 mg/L (2*S*)-naringenin was added. (B) Effects of overexpressing stress resistance genes on (2*S*)-sakuranetin production and cell growth when 500 mg/L (2*S*)-naringenin was added. (C) Effects of overexpressing stress resistance genes on (2*S*)-sakuranetin production and cell growth when 600 mg/L (2*S*)-naringenin was added. (D) Spot assay of strain tolerance ability to (2*S*)-naringenin. (E) The growth status and production of (2*S*)-sakuranetin when *rpsQ*^His31Pro^ and *secB* were co-expressed with *rpoS*. ****P < 0.0001.

The (2*S*)-sakuranetin titer decreased with increasing (2*S*)-naringenin concentrations, which may due to the low tolerance of strain to (2*S*)-naringenin. Ribosomal subunits (RpsQ, RpsQ^His31Pro^, and RpsL [26]), molecular chaperones (SecB [27], Ycdy [28], Nfua [29], and Yajl [30]), sRNAs (RpoS [31], ProQ [32], NusB [33], AcrR [34], and Asr [35]), and the global regulatory transcription factor CRP [36], which can participate in cellular stress response, were expressed to select the most suitable gene to improve strain tolerance to (2*S*)-naringenin. (2*S*)-Naringenin was supplemented in batches to a final concentration of 400 mg/L, 500 mg/L and 600 mg/L (substrate was added as described in Section 3.4). The (2*S*)-sakuranetin titer with strain NS21, NS23 and NS24 was obviously increased at different (2*S*)-naringenin concentrations compared with the strain 7-FOMT-3 (Fig. 6A–C); when 500 mg/L (2*S*)-naringenin was added, the production of (2*S*)-sakuranetin increased by 108.56%, 122.63%, and 104.26% compared with the strain 7-FOMT-3, respectively.

Strain NS21, NS23 and NS24 harbor plasmid pET-28a (+)-*PfOMT3*-*rpsQ*^His31Pro^, pET-28a (+)-*PfOMT3*-*rpoS* and pET-28a (+)-*PfOMT3-secB*, respectively. Genes *rpsQ*, *rpoS* and *secB* can relieve cell pressure to a certain extent; the gene *rpsQ* can reduce protein mistranslation [37] and *rpsQ*^His31Pro^ has been proven to have better stress tolerance [26], the gene *secB* can prevent protein aggregation [38] and promote normal transport of protein [39,40], and the gene *rpoS* can increase energy metabolism under stress [41] and regulate protein expression in normal state [42]. To evaluate intuitively the tolerance ability of strain containing *rpsQ*^His31Pro^, *rpoS* and *secB* to (2*S*)-naringenin, spot assay was conducted. Strain NS21, NS23 and NS24 all grew better than strain 7-FOMT-3 on solid LB plates containing (2*S*)-naringenin (Fig. 6D). Then, *rpsQ*^His31Pro^ and *secB* were expressed with *rpoS*; however, the strain harboring the pET-28a (+)-*PfOMT3*-*rpoS*-*rpsQ*^His31Pro^ plasmid (NS32) accumulated less (2*S*)-sakuranetin, resulting in a 36.62% and 40.62% decrease compared with strain NS21 and NS23, respectively, when added 500 mg/L (2*S*)-naringenin (Fig. 6E), which may due to the imbalance of cell metabolism. Therefore, the gene *rpoS* and the genes combination *rpoS*-*secB* were selected for expression with methylation-enhancing genes.

### 3.6 Fermentation process optimization to improve (2*S*)-sakuranetin production

**Fig. 7:**
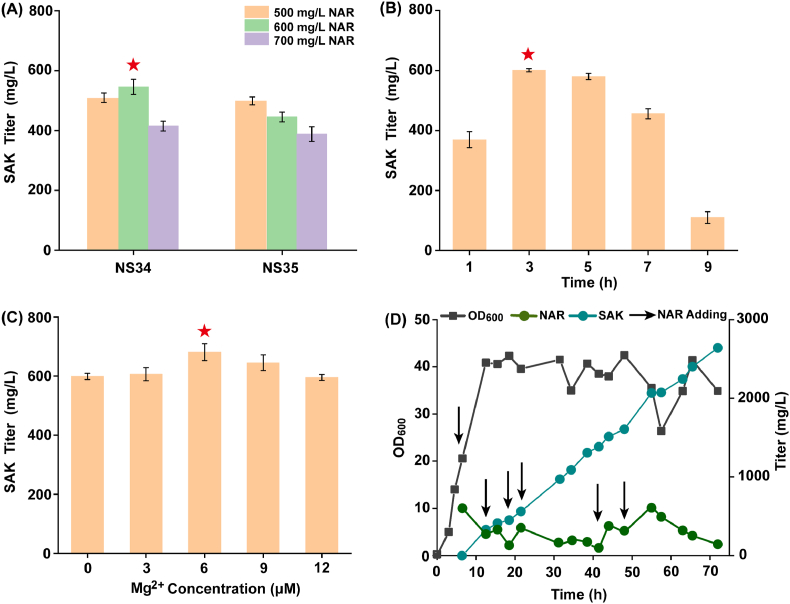
Fermentation process optimization to improve (2*S*)-sakuranetin production. (A) The accumulation of (2*S*)-sakuranetin when *rpoS* and *secB* were co-expressed with methylation-strengthening genes. (B) Effects of different (2*S*)-naringenin addition time on the production of (2*S*)-sakuranetin. The final concentration of (2*S*)-naringenin was 700 mg/L. (C) Effects of different Mg^2+^ levels on the production of (2*S*)-sakuranetin. (2*S*)-Naringenin was first added after IPTG was added for 3 h, and different concentrations of Mg^2+^ were added at the same time. The final concentration of (2*S*)-naringenin was 700 mg/L. (D) Strain NS34 was fermented in a 5-L bioreactor, and (2*S*)-naringenin was added in batches to a final concentration of 4 g/L.

The production of (2*S*)-sakuranetin was further improved when substrate tolerance enhancement genes *rpoS*, *secB* and methylation enhancement genes were co-expressed. The result showed strain NS34 accumulated the highest titer of (2*S*)-sakuranetin; 545.98 mg/L of (2*S*)-sakuranetin was obtained when 600 mg/L (2*S*)-naringenin was added (Fig. 7A), which was 95.43% higher than the optimal strain improving cell substrate tolerance alone, and was 131.71% higher than the optimal strain enhancing methylation process alone.

To determine the optimal substrate addition time point, (2*S*)-naringenin was first supplemented after IPTG was added for 1 h, 3 h, 5 h, 7 h and 9 h (final concentration was 700 mg/L), respectively. The result showed that the optimal substrate addition time was 3 h after the addition of IPTG (Fig. 7B). In addition, considering that Mg^2+^ can activate the leaving group (ATP and giving phosphate) in the biosynthesis of SAM [43] and that SAM synthetase requires Mg^2+^, different concentrations of Mg^2+^ were added. 681.44 mg/L of (2*S*)-sakuranetin was obtained when added 6 mM Mg^2+^ (Fig. 7C). Strain NS34 was used for scale-up culturing in a 5-L bioreactor, and 2642.38 mg/L of (2*S*)-sakuranetin was obtained at 70 h from 4 g/L (2*S*)-naringenin (Fig. 7D).

## 4 Discussion

Previous studies have revealed that *E. coli* has the ability to express SAM-dependent F7-OMTs. In this study, PfOMT3 was selected as the optimal F7-OMT to convert (2*S*)-naringenin to (2*S*)-sakuranetin. Given that exogenously supplied of methionine could improve (2*S*)-sakuranetin titer and that the conversion efficiency decreased with increasing concentration of (2*S*)-naringenin, methyl donor and cell tolerance to substrate were considered as limiting factors for (2*S*)-sakuranetin biosynthesis. Experiments were performed to upregulate methionine, cofactor ATP and PLP content to enhance methylation process, and to select the best molecular chaperone, sRNA or ribosomal subunits to improve strain tolerance to (2*S*)-naringenin. No previous studies have focused on enhancing the methylation process and improving the bacterial resistance to achieve the efficient synthesis of (2*S*)-sakuranetin.

The methyl donor SAM, biosynthesized from methionine and ATP by SAM synthetase (encoded by *metK* in *E. coli*), plays an important role in methylation process. As the direct precursor of SAM, methionine content was enhanced by upregulating key enzymes MetA [44] and CysE [45] in methionine biosynthesis pathway, and the production of (2*S*)-sakuranetin increased by 50.26% compared with strain 7-FOMT-3. (2*S*)-Sakuranetin titer decreased when *metK* was overexpressed, which may due to the overproduction of *metK* would consume large amount of ATP and cause a methionine deficiency [46], resulting in the imbalance of metabolic flux.

Methylation proceess was further upregulated by regulating ATP and NADPH contents, which are important cofactors for SAM biosynthesis. Previous studies proved that *ydaO* from *B. subtilis* can dynamically regulate ATP content in *E. coli* [47,48]. The accumulation of (2*S*)-sakuranetin increased by 162.38% in the *ydaO*-expressing strain compared with strain 7-FOMT-3. *POS5* is known to be involved in NADPH regeneration in *S. cerevisiae* and can increase the intracellular NADPH availability in *E. coli* [49]. (2*S*)-Sakuranetin titer increased when *POS5* was expressed in strain 7-FOMT-3. However, the titer of (2*S*)-sakuranetin decreased when *POS5* was co-expressed with *metA*, *cysE*, *ydaO*, and the PLP biosynthesis genes. Considering that the disturbance of NADPH pool would trigger the redistribution of metabolic flux in *E. coli* [50], it was speculated that the redox and metabolic flux imbalance caused the decrease of (2*S*)-sakuranetin titer. In addition, the extreme metabolic burden of overexpression too many foreign genes may also result in the decline of (2*S*)-sakuranetin production.

Enzymes involved in the transsulfuration pathway utilize PLP as cofactor [51], whether the increase of PLP content could enhance the synthesis of (2*S*)-sakuranetin was explored. *RFC4* and *RPS18B* are known can increase intracellular pyridoxine and pyridoxamine concentrations [25], and pyridoxine and pyridoxamine can be converted into PLP [52]. Given that genes involved in the transsulfuration pathway have a high homology between *E. coli* and *S. cerevisiae* [53], *RPS18B* and *RFC4* from *S. cerevisiae* were heterologously expressed in *E. coli*. The (2*S*)-sakuranetin titer increased by 180.72% when *SNZ3*, *RPS18B*, and *RFC4* were co-expressed compared with the strain 7-FOMT-3. This result provides a valuable insight to biosynthesize other methylated products in *E*. *coli*, and demonstrates the significance of PLP in methylation process.

Flavonoids are known to have antimicrobial properties; they can inhibit DNA gyrase [15], alter the cell membrane permeability, and change cellular morphology [54], which would affect the metabolism and function of cells. In addition, (2*S*)-sakuranetin titer decreased with increasing concentrations of (2*S*)-naringenin. Therefore, substrate resistance was considered as another limiting factor. Genes that can enhance cell tolerance to (2*S*)-naringenin were identified in this study. Strains expressing *rpsQ*^His3^^^^1^^^^Pro^, *rpoS*, and *secB* showed better performance on (2*S*)-sakuranetin production and cell growth. The spot assay reflected more intuitively that RpsQ^His3^^^^1^^^^Pro^, RpoS, and SecB protein could improve the (2*S*)-naringenin tolerance of host cells. This result provides an idea to alleviate cell stress in biosynthesizing other natural products with antibacterial effect in *E*. *coli*, and the selected genes may also be used to relieve cells pressure in the conversion of other flavonoids.

In conclusion, an efficient (2*S*)-sakuranetin-producing strain was constructed by F7-OMTs selection, methylation process enhancement, cell resistance improvement and fermentation process optimization. The titer of (2*S*)-sakuranetin reached 2642.38 mg/L in a 5-L bioreactor, which is the highest titer reported to date. Given that flavonoid *O*-methyltransferases (FOMTs) have substrate specificity [55], other valuable methylated flavonoids might be obtained efficiently based on selecting specific FOMTs and adopting strategy of this study.

## CRediT authorship contribution statement

**Qiumeng Sun:** Methodology, Investigation, Formal analysis, Writing - original draft, Writing - review & editing. **Song Gao:** Formal analysis, Writing - review & editing. **Shiqin Yu:** Methodology, Supervision. **Pu Zheng:** Methodology, Supervision. **Jingwen Zhou:** Methodology, Supervision, Funding acquisition, Writing - review & editing.

## Declaration of competing interest

The authors declare that they do not have any financial or commercial conflict of interest in connection with the work submitted.
