# Supplementary material for: Production of (2S)-sakuranetin from (2S)-naringenin in Escherichia coli by strengthening methylation process and cell resistance
Source: Synth Syst Biotechnol. 2022 Aug 3;7(4):1117–25. doi: 10.1016/j.synbio.2022.07.004 (PMC9399173; doi:10.1016/j.synbio.2022.07.004)
Supplement: Multimedia component 1 [file mmc1.docx]

**Production of (2*S*)-sakuranetin from (2*S*)-naringenin in *Escherichia coli* by strengthening methylation process and cell resistance**

Qiumeng Sun^1,2,3,4^, Song Gao^1,2,3,4^, Shiqin Yu^1,2,4^, Pu Zheng^2*^, Jingwen Zhou^1,2,3,4*^

^1^ Science Center for Future Foods, Jiangnan University, 1800 Lihu Road, Wuxi, Jiangsu 214122, China;

^2^ Key Laboratory of Industrial Biotechnology, Ministry of Education and School of Biotechnology, Jiangnan University, 1800 Lihu Road, Wuxi, Jiangsu 214122, China;

^3^ Engineering Research Center of Ministry of Education on Food Synthetic Biotechnology, Jiangnan University, 1800 Lihu Road, Wuxi, Jiangsu 214122, China;

^4^ Jiangsu Province Engineering Research Center of Food Synthetic Biotechnology, Jiangnan University, Wuxi 214122, China.

* Correspondence to:

Pu Zheng, Jingwen Zhou,

Science Center for Future Foods, Jiangnan University, 1800 Lihu Road, Wuxi, Jiangsu 214122, China.

Phone: +86-510-85914371, Fax: +86-510-85914371

E-mail: zhengpu@jiangnan.edu.cn, zhoujw1982@jiangnan.edu.cn

The process for plasmids construction

PCR and DNA fragments ligations were performed according to manufacturer’s instructions. All fragments were amplified by High-fidelity Phusion DNA polymerase (Vezyme, Nanjing, China). DNA fragments ligations were performed using the Seamless Cloning kit (Sangon, Shanghai, China). To construct plasmid pCDFDuet-*metA*-*cysE*, primers pCDF-R/pCDF-M1-F were used to linearize pCDFDuet-1; primers metA-M1-F/metA-cysE-R and cysE-metA-F/cysE-M1-R were used to amplify *metA* and *cysE* from *E. coli* genomic DNA. The *metA* and *cysE* fragments were cloned into MCS1 of pCDFDuet-1. Primers ydaO-M2-F/ydaO-M2-R and pCDF-F/pCDF-M2-R was used to amplify *ydaO* from *B. subtilis* genomic DNA and linearize pCDFDuet-*metA*-*cysE*. Then *ydaO* fragments were cloned into MCS2 of pCDFDuet-*metA*-*cysE*, resulting in pCDFDuet-*metA*-*cysE-ydaO*. The construction methods of other plasmids were same to the above. All primers used in this study are listed in Table S1.

Table S1 Primers used for constructing plasmid

| **Primers** | **Sequences** |
| --- | --- |
| pCDF-M1-F | AAGCTTGCGGCCGCAT |
| pCDF-R | GCCCATGGTATATCTCCTTATTAAAGTTAAAC |
| metA-M1-F | GTTTAACTTTAATAAGGAGATATACCATGGGCatgccgattcgtgtgccgg |
| metA-cysE-R | caatttccagttcttcacacgacatggtatatctccttttaatccagcgttggattcatgtgc |
| pCDF-F | ACCTAGGCTGCTGCCAC |
| pCDF-M2-R | TAACATATGTATATCTCCTTCTTATACTTAACTAATATACTAAGATGG |
| metK-M2-F | GAAGGAGATATACATATGTTAATGGCAAAACACCTTTTTACGTCCGA |
| metK-M2-R | GTGGCAGCAGCCTAGGTTTACTTCAGACCGGCAGCATCG |
| ydaO-M2-F | CTTAGTATATTAGTTAAGTATAAGAAGGAGATATACATATGTTAATGTATCATTCAATCAAACGTTTTTTGATTGGG |
| ydaO-M2-R | GCTCAGCGGTGGCAGCAGCCTAGGTTTACTTTTTAAAATGATACGGCAGTGTGGC |
| ydaO-metK-R | TTACTTTTTAAAATGATACGGCAGTGTGGCA |
| metK-ydaO-F | CGTATCATTTTAAAAAGTAAAAGGAGATATACCATGGCAAAACACCTTTTTACGTCCG |
| pACY-M1-F | AAGCTTGCGGCCGCATAATG |
| pACY-R | GCCCATGGTATATCTCCTTATTAAAGTTAAAC |
| SNZ3-M1-F | CTTTAATAAGGAGATATACCATGGGCATGTCAGAATTCAAGGTTAAAACTGGGC |
| SNZ3-M1-R | CTTAAGCATTATGCGGCCGCAAGCTTCTACCATCCGATTTCAGAAAGTCTTGCAC |
| RPS18B-M1-F | AATAAGGAGATATACCATGGGCATGTCTTTAGTTGTACAAGAACAAGGTTCCTTC |
| RPS18B-M1-R | CATTATGCGGCCGCAAGCTTTTAAGCTCTTCTTCTACCAGTGGTCTTGG |
| RFC4-M1-F | CTTTAATAAGGAGATATACCATGGGCATGTCCAAAACTTTATCTTTGCAACTTCC |
| RFC4-M1-R | GCATTATGCGGCCGCAAGCTTTCAGGCTTTATTATTTAGTTTATGAATTTTCGCTAAC |
| PACYC-M2-R | CATATGTATATCTCCTTCTTATACTTAACTAATATACTAAGATGGGG |
| PACYC-F | TTAACCTAGGCTGCTGCCACC |
| SNZ3-RPS18B-R | GTTCTTGTACAACTAAAGACATGGTATATCTCCTTCTACCATCCGATTTCAGAAAGTCTTGCAC |
| RPS18B-SNZ3-F | ATGTCTTTAGTTGTACAAGAACAAGGTTCCTTC |
| RPS18B-M1-R | CATTATGCGGCCGCAAGCTTTTAAGCTCTTCTTCTACCAGTGGTCTTGG |
| RFC4-M2-F | AGTATAAGAAGGAGATATACATATGATGTCCAAAACTTTATCTTTGCAACTTCCATG |
| RFC4-M2-R | GGTGGCAGCAGCCTAGGTTAATCAGGCTTTATTATTTAGTTTATGAATTTTCGCTAACATACTAG |
| pET28(a)-Pf-F | CACCACCACCACCACCAC |
| pET28(a)-Pf-R | TTATTTGTACAGTTCCATGATCCAGGTAGAG |
| POS5-Pf-F | CTGGATCATGGAACTGTACAAATAAAAGGAGATATACCATGAGTACGTTGGATTCACATTCCCT |
| POS5-Pf-R | CAGTGGTGGTGGTGGTGGTGTTAATCATTATCAGTCTGTCTCTTGGTCAGCC |
| rpsL-Pf-F | CCTGGATCATGGAACTGTACAAATAAAAGGAGATATACCATGGCAACAGTTAACCAGCTGG |
| rpsL-Pf-R | CTCAGTGGTGGTGGTGGTGGTGTTAAGCCTTAGGACGCTTCACGC |
| rpsQ-Pf-F | CTGGATCATGGAACTGTACAAATAAAAGGAGATATACCATGACCGATAAAATCCGTACTCTGCAAG |
| rpsQ-Pf-R | CAGTGGTGGTGGTGGTGGTGTTACAGAACCGCTTTCTCTACAACGC |
| rpsL ^His31Pro^-F | GTTTTGTGAAACCCCCGATCTACGGTAAATTCATCAAGCGTAC |
| rpsL ^His31Pro^-R | GTACGCTTGATGAATTTACCGTAGATCGGGGGTTTCACAAAAC |
| crp-Pf-F | CTGGATCATGGAACTGTACAAATAAAAGGAGATATACCATGGTGCTTGGCAAACCGC |
| crp-Pf-R | CAGTGGTGGTGGTGGTGGTGTTAACGAGTGCCGTAAACGACGATG |
| rpoS-Pf-F | GGATCATGGAACTGTACAAATAAAAGGAGATATACCATGAGTCAGAATACGCTGAAAGTTCATGATT |
| rpoS-Pf-R | CAGTGGTGGTGGTGGTGGTGTTACTCGCGGAACAGCGCTT |
| secB-Pf-F | CTGGATCATGGAACTGTACAAATAAAAGGAGATATACCATGTCAGAACAAAACAACACTGAAATGGCA |
| secB-Pf-R | GATCTCAGTGGTGGTGGTGGTGGTGTCAGGCATCCTGATGTTCTTCAG |
| nufA-Pf-F | GATCATGGAACTGTACAAATAAAAGGAGATATACCATGATCCGTATTTCCGATGCTGC |
| nufA-Pf-R | CAGTGGTGGTGGTGGTGGTGTTAGTAGTAGGAGTGTTCGCCGCG |
| yajL-Pf-F | GGATCATGGAACTGTACAAATAAAAGGAGATATACCATGAGCGCATCGGCACTG |
| yajL-Pf-R | CAGTGGTGGTGGTGGTGGTGCTACTCGTAATAATTATAAATCCCTGCCGCC |
| ycdY-Pf-F | GGATCATGGAACTGTACAAATAAAAGGAGATATACCATGAACGAGTTTTCTATCCTCTGTCGTG |
| proQ-Pf-R | CAGTGGTGGTGGTGGTGGTGTTATTCTTCAGAATCTTCTTCCAGCTCGTCC |
| proQ-Pf-F | GGATCATGGAACTGTACAAATAAAAGGAGATATACCATGGAAAATCAACCTAAGTTGAATAGCAGTAAAGAAG |
| proQ-Pf-R | CAGTGGTGGTGGTGGTGGTGTCAGAACACCAGGTGTTCTGCG |
| proQ-Pf-F | CTGGATCATGGAACTGTACAAATAAAAGGAGATATACCGTGAAACCTGCTGCTCGTCG |
| proQ-Pf-R | CAGTGGTGGTGGTGGTGGTGTCACTTTTTGTTAGGGCGAATCACAGG |
| acrR-Pf-F | CTGGATCATGGAACTGTACAAATAAAAGGAGATATACCATGGCACGAAAAACCAAACAAGAAGC |
| acrR-Pf-R | CAGTGGTGGTGGTGGTGGTGTTATTCGTTAGTGGCAGGATTACGAAGCG |
| asr-Pf-F | GGATCATGGAACTGTACAAATAAAAGGAGATATACCATGAAAAAAGTATTAGCGCTGGTTGTTGC |
| asr-Pf-R | CAGTGGTGGTGGTGGTGGTGTTACGCTGCGGGTTGTGCA |
| rpsQ-rpoS-R | TTACAGAACCGCTTTCTCTACAACGC |
| rpoS-rpsQ-F | CGTTGTAGAGAAAGCGGTTCTGTAAAAGGAGATATACCATGAGTCAGAATACGCT |
| secB-rpoS-R | TCAGGCATCCTGATGTTCTTCAGTACC |
| rpoS-secB-F | GAAGAACATCAGGATGCCTGAAAGGAGATATACCATGAGTCAGAATACGCTGAAAGTTCATGATTTAAATG |
